# Transient block of interatrial epicardial connection during right-sided pulmonary vein encircling demonstrated by high-density 3-dimensional mapping

**DOI:** 10.1016/j.hrcr.2023.05.013

**Published:** 2023-05-27

**Authors:** Kentaro Hayashi, Takeshi Kitamura, Masayuki Ohta, Naoki Masuda, Nobuhiko Ogata, Takaaki Isshiki

**Affiliations:** Department of Cardiology, Ageo Central General Hospital, Ageo-city, Japan

**Keywords:** Epicardial connection, Pulmonary vein isolation, Catheter ablation, 3D mapping, Pulmonary vein reconnection

**Key Teaching Points**

• An interatrial epicardial connection (EC) between the right-sided pulmonary vein (PV) and right atrium is one of the mechanisms that can preclude PV isolation.
• Circumferential PV isolation may transect EC by accident during the first encircling of right-sided PVs, but sometimes its effect may be transient and lead to PV reconnection.
• A careful reevaluation of right-sided PV potential after a waiting time is necessary, and modulation of application at the right anterior carina to the bottom of the right inferior PV where the EC transects the PV isolation circle should be considered.

## Introduction

Circumferential pulmonary vein isolation (CPVI) is the cornerstone of catheter ablation for atrial fibrillation (AF),^1^ but it is not always achieved after the initial encircling of pulmonary veins (PVs). Studies found that the existence of epicardial connection (EC) via intercaval fibers, connecting right-sided PVs (RPV) and right atrium (RA), can preclude the isolation of these PVs.^2^, ^3^, ^4^, ^5^ A previous report indicated that an anterior ablation line of the pulmonary vein isolation (PVI) circle for the isolation of RPVs intersects a theoretical pathway of EC and transiently eliminate EC based on changes in PV potential sequences.^6^ In the present case, a transient elimination of interatrial EC and reconnection via EC during right-sided CPVI were revealed by a dramatic change in activation patterns in RA using high-density 3D mapping.

## Case report

**Figure 1:**
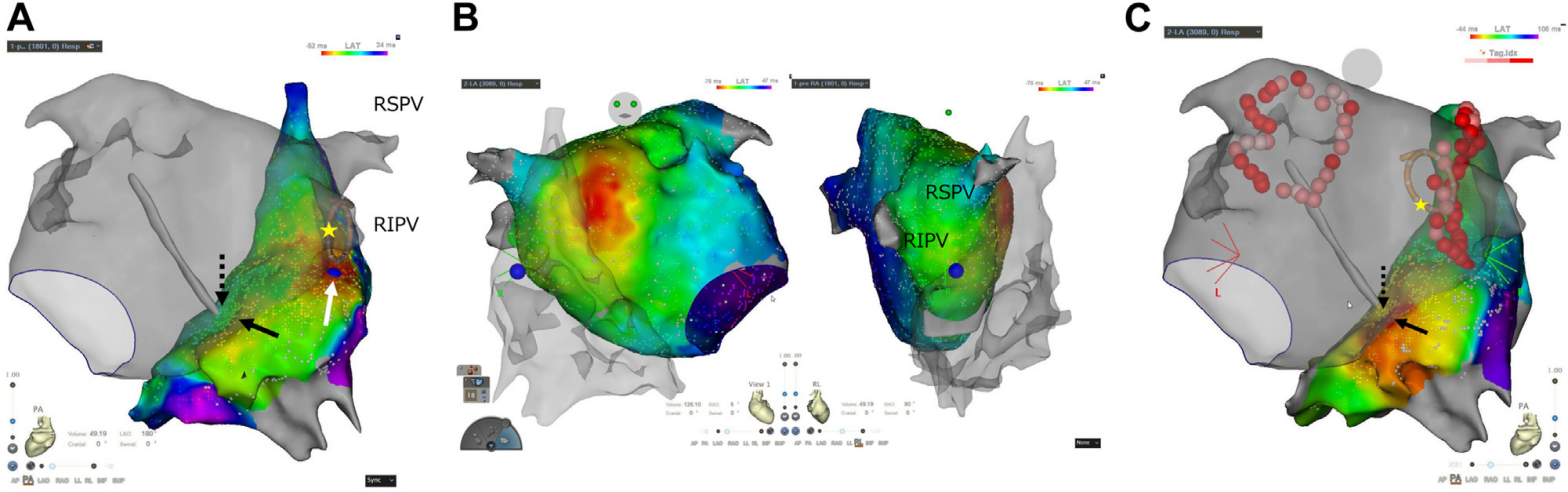
**A:** Activation map of right atrium (RA) before pulmonary vein isolation (PVI) under pacing from the right inferior pulmonary vein (RIPV) at the carina posterior part to highlight the epicardial connection (EC) between the right-sided pulmonary vein (RPV) and RA. The high-resolution map revealed an interatrial conduction from left atrium (LA) to RA via EC at the posterior wall of the RA (*white arrow*) and interatrial septum (*black arrow*). The blue tag indicates the earliest activation site in the RA under pacing from the RIPV. The yellow star sign indicates pacing site. The dotted arrow indicates the transseptal puncture site depicted by fast anatomical map (FAM) using an ablation catheter. **B:** The LA activation map under high RA pacing shows no evidence of conduction breakthrough from RA to LA proximal to the RPV. **C:** The RA activation map after PVI revealed an interatrial conduction from LA to RA only via the interatrial septum (black arrow), suggesting that the anterior part of PVI circle intersected EC and eliminated its conduction by chance. The yellow star sign indicates the pacing site. The dotted arrow indicates the transseptal puncture site depicted by FAM using an ablation catheter.

A 75-year-old female patient with mitral regurgitation, chronic heart failure, hypertension, and symptomatic short persistent AF was referred to our institution for AF ablation after pilsicainide therapy failure. A mapping procedure was performed using a 3-dimensional navigation system (CARTO 3; Biosense Webster, Inc, Diamond Bar, CA) and Pentaray catheter (Biosense Webster, Inc). Before PVI, to expose the EC between RPVs and RA, an activation map of the RA was created under pacing from the RPV at the carina posterior part to avoid far-field RA capture. A left atrium (LA) activation map during pacing from the high RA was described as well. The RA activation map revealed an interatrial conduction from LA to RA via EC at the posterior wall of the RA (Figure 1A, white arrow) and interatrial septum (Figure 1A, black arrow), while there was no evidence of conduction breakthrough from RA to LA close to the RPV in the LA activation map under high RA pacing (Figure 1B).

CPVI was performed using point-by-point radiofrequency (RF) applications with a ThermoCool SmartTouch irrigated tip contact force–sensing ablation catheter (Biosense Webster, Inc). After successfully isolating left-sided PVs, CPVI for RPVs was performed during high RA pacing. An RF energy with a power setting of 35–40 W and ablation index of 400–450 was delivered from the posterior to the anterior part of RPVs. First-pass CPVI was achieved, and bidirectional block was confirmed. Then, an RA activation map was obtained under left posterior wall pacing just outside the RPV circle close to the pacing site in the initial RA map.

**Figure 2:**
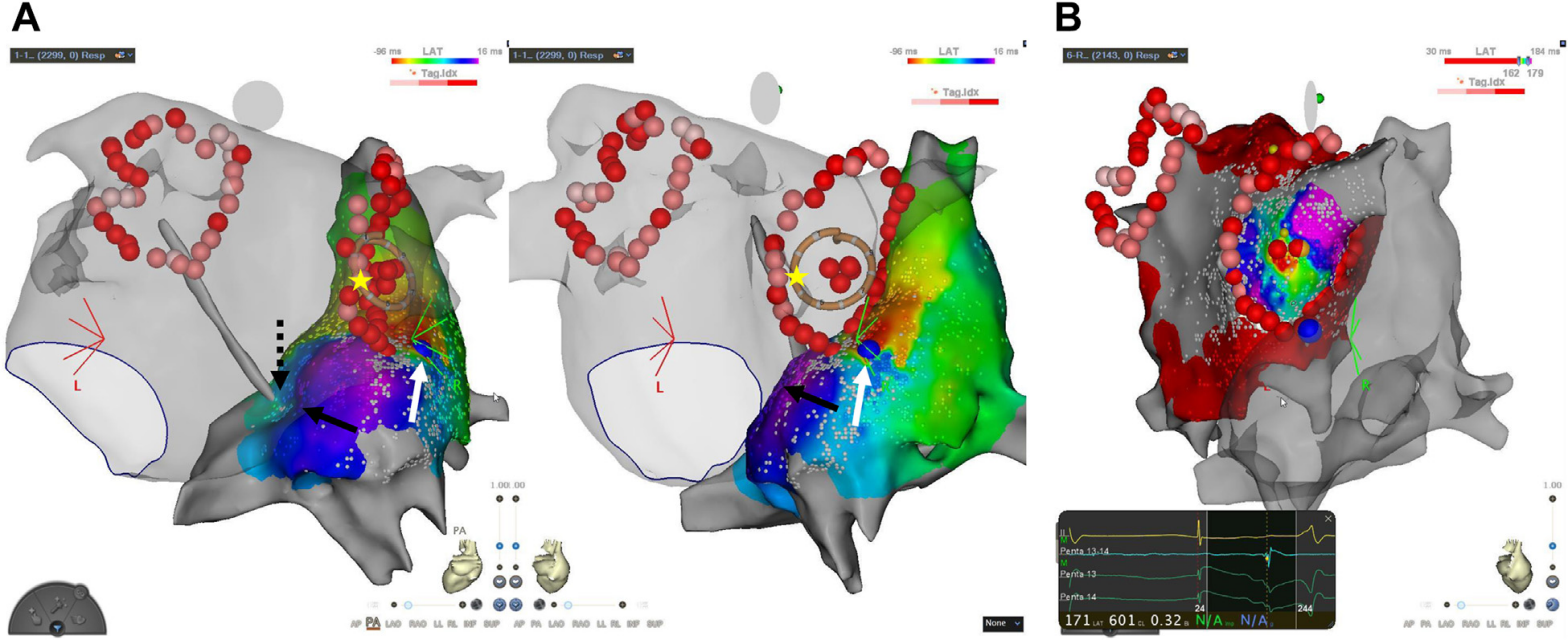
**A:** The RA activation map after PV reconnection described under pacing from inside the RIPV revealed an interatrial conduction from LA to RA via only EC (*white arrow*), which was identical to that before PVI (*white arrow* in Figure 1A). The activation from the interatrial septum disappeared (*black arrow*) because the PVI circle was completed, and the conduction from PV to LA antrum was blocked. The yellow star sign indicates the pacing site. The blue tag indicates the earliest activation site in the RA under pacing from the RIPV. The dotted arrow indicates the transseptal puncture site depicted by FAM using an ablation catheter. **B:** The LA activation map after PV reconnection under coronary sinus pacing revealed that the earliest activation site in the PVI circle was the RPV carina, and LA antrum–PV conduction block was confirmed. The red dots indicate the ablation sites. Abbreviations as in Figure 1.

The RA activation map revealed an interatrial conduction from LA to RA only via the interatrial septum (Figure 1C, black arrow), suggesting that the anterior part of the RPV circle intersected the EC and eliminated its conduction by chance. After 15 minutes of waiting time, PV reconnection was observed at the RPV. The pacing maneuvers reported by Hasebe and colleagues^2^ to differentiate EC from residual conduction gaps on the CPVI line suggest that the reconnection was via EC (time interval between RPV and the posterior antrum of the right superior PV just outside the CPVI line under RA pacing and coronary sinus pacing was 42 ms). The RA activation map constructed under pacing from inside the right inferior pulmonary vein (RIPV) revealed an interatrial conduction from LA to RA only via EC (Figure 2A, white arrow), which was identical to that before PVI (Figure 1A, white arrow). The activation from the interatrial septum disappeared (Figure 2A, black arrow) because the PVI circle was completed, and the conduction from PV to LA antrum was blocked. Subsequently, an LA activation map under coronary sinus pacing was made, and the earliest activation site in the PVI circle was at the RPV carina; LA antrum–PV conduction block was confirmed. The success ablation site was 10 mm away from the PVI circle (Figure 2B). The application at the RPV carina breakthrough site via EC resulted in the elimination of PV potentials, and bidirectional block was confirmed after 30 minutes of waiting and adenosine triphosphate infusion.

## Discussion

To the best of our knowledge, this is the first report demonstrating transient block of interatrial EC during RPV encircling based on high-density 3D mapping. This includes RA activation maps taken before and after PV applications to identify electrical connection sites between RA and LA with and without EC. In this case, the RPVs were successfully isolated in the first CPVI, but they reconnected during the procedure. The activation map obtained during pacing from the RIPV showed that the posterior wall of the RA via EC and the interatrial septum were the electrical connection from LA to RA before ablation. After the first CPVI, the electrical connection from LA to RA changed to via the interatrial septum only; then, the activation pattern varied to via EC only when the RPV reconnection happened. In the LA map under high RA pacing before CPVI, there was no obvious EC in the RPV carina; however, after the reconnection of the RPV, an apparent carina breakthrough was observed.

The activation pattern from RA to LA depends on the pacing site^5^ and conduction velocity of the Bachmann bundle and other pathways. In the present case, the conduction from RA to LA via the Bachmann bundle was faster and closer to the pacing site than that via EC; thus, the conduction via EC was masked by the Bachmann bundle conduction before ablation.

**Figure 3:**
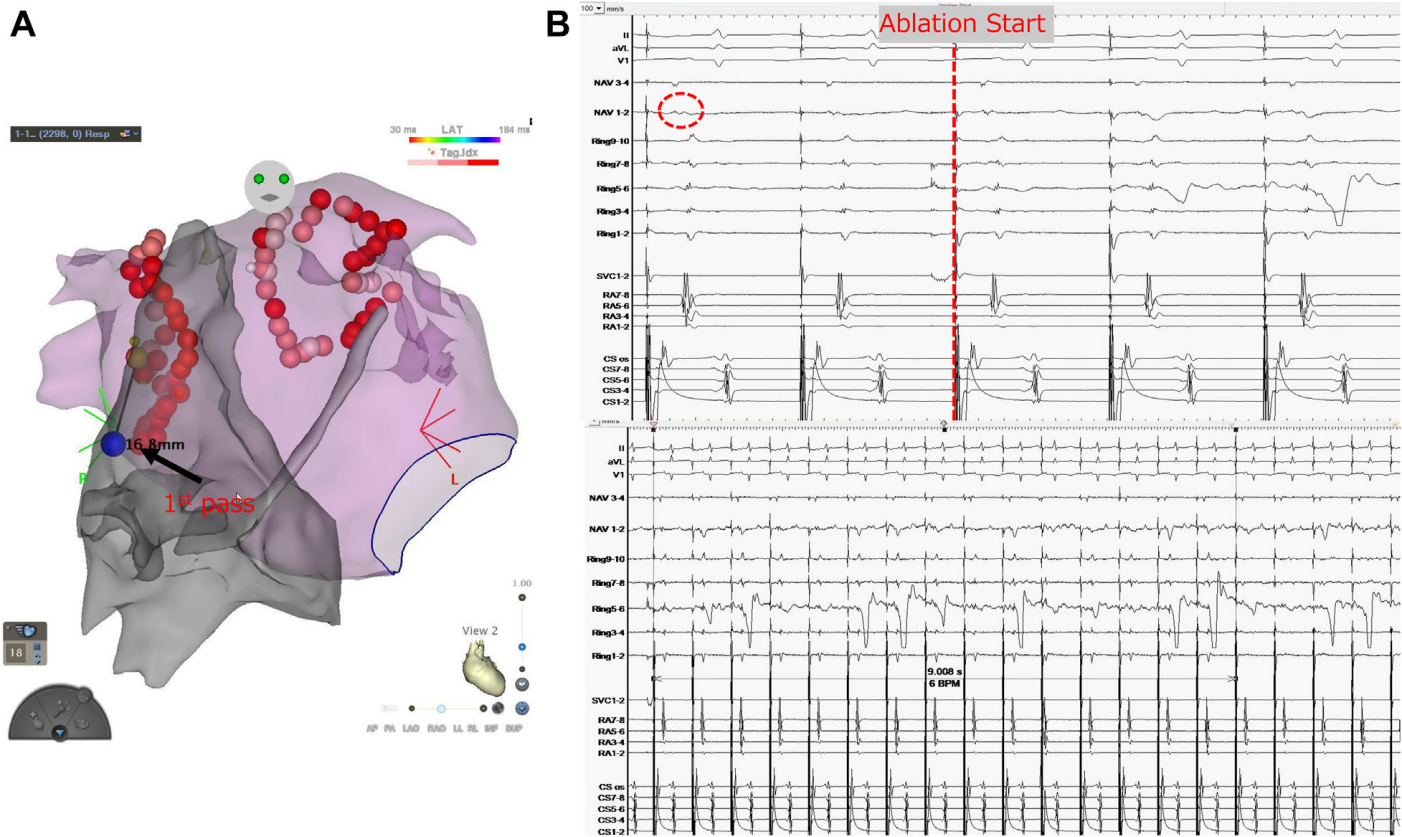
**A:** The theoretical pathway of the EC can be imaged by connecting the earliest activation sites of the RA under pacing from the RPV and the site of RPV carina breakthrough. The 32nd application in the firsr encircling isolating the RPV was closest to the theoretical EC pathway (*black arrow*). The blue tag indicates the earliest activation site in the RA before ablation under pacing from the RPV, while the yellow tag indicates the earliest activation site in the PVI circle under coronary sinus pacing after RPV reconnection (RPV carina breakthrough). **B:** Before the 32nd application in the first RPVI, the atrial potential in the ablation catheter was very small (0.08 mV, dotted circle), and 9 seconds was required for isolation. This phenomenon suggests that this application transiently blocked the conduction via the EC from the endocardium. Abbreviations as in Figure 1.

We obtained 3089 points in LA mapping; however, there was some limitation in the density of mapping in the PV carina to elucidate the existence of EC. The theoretical pathway of EC in the present case can be imaged by connecting the earliest activation sites of RA under pacing from the RPV and the site of the right PV carina breakthrough (Figure 3A). While the 32nd application in the first CPVI isolated the RPVs, the atrial potential before the application was very small (0.08 mV), and it took 9 seconds to achieve isolation (black arrow, Figure 3A). This suggests that the application may have transiently blocked the conduction via the EC pathway from the endocardium because this site was closest to the theoretical EC pathway, and the distance between that and the ablation site was 5 mm.

Previous anatomical studies clearly revealed muscular bridges connecting the RPVs and RA.^3^ Studies also showed that 6.7%–15.0% of patients were considered to have EC between the RPVs and RA after PV applications from electrophysiologic aspects.^2^^,^^4^ If EC were incidentally eliminated by RF applications from the endocardium during CPVI when the ablation circle was close to the EC, this phenomenon might create an insufficient lesion at the EC and could be a risk for acute or chronic PV reconnection. Moreover, in some cases, CPVI may transect EC during the first encircling of RPVs by accident.

Although the ablation strategy for EC has not been well established yet, physicians should be aware of the risk of PV reconnection via EC after CPVI to achieve a durable PVI. A careful reevaluation of RPV potentials is necessary after a waiting period. The modulation of application power and time from the anterior carina to the bottom of the RIPV should be considered, where EC was transected by the PVI circle. High-power short-duration applications may be unsuitable for achieving a durable effect on EC because this mode creates a shallower lesion.^7^ An overly broad isolation range in the anterior part of the RPV is also unfavorable for the EC transection.

## Conclusion

The EC in the present case was considered to be accidentally and transiently blocked by CPVI. Physicians should be aware that CPVI can transect EC by transmural lesion formation and cause acute or chronic PV reconnection via the EC if the RF energy is insufficient to durably block it.
